# Clinical Neuropathology image 6-2014: Corpora amylacea replacing cornu ammonis (CACA)

**DOI:** 10.5414/NP300831

**Published:** 2014-10-23

**Authors:** Gabor G. Kovacs, Daniele Risser

**Affiliations:** 1 Institute of Neurology and; 2 Department of Forensic Pathology, Medical University of Vienna, Vienna, Austria

**Keywords:** corpora amylacea, τ-astrogliopathy, cornu ammonis, hippocampus

## Abstract

Not available.

Corpora amylacea (CA) are globular bodies usually 10 – 50 µm in diameter mainly made up of glucose polymers (polyglucosans), which stain strongly with periodic acid-Schiff (PAS) staining. They are commonly seen in different subependymal and subpial regions, fornix, olfactory tract, posterior column of the spinal cord, and velum medullare anterius [1]. Large amounts of CA have been reported in a subset of patients with temporal epilepsy specimens, even where unusual and extensive amounts of CA replaced the pyramidal layers of the cornu ammonis [2]. However, others concluded this to be a pathological response to neuronal cell loss with no clear clinical and quantitative hippocampal MRI correlates [3]. 

We present here a case where extensive amounts of CA were detected. A 73-year-old man living in a nursery home fell and suffered head trauma. We do not have any documentation of follow-up neurological examinations of the patient. Following neurosurgical intervention, repeated cranial CT revealed progressively increasing subdural and subarachnoidal bleedings with severe brain edema and the patient died. Forensic pathological examination was performed and blocks of the cerebellum and hippocampus formation were sampled to evaluate the degree of hypoxic/ischemic damage. There was severe neuronal loss in the cornu ammonis associated with abundant PAS-positive CA replacing the pyramidal layers (Figure 1A, B). Mostly the smaller CA showed GFAP immunoreactivity hugging the globular bodies (Figure 1C). Axons seemed to be distorted in SMI-31 and -32 neurofilament stainings (Figure 1D). Interestingly, immunostaining for phosphorylated TDP-43 showed prominent immunoreactivity in the CA1 (Figure 1E) representing a neurodegenerative process. Furthermore, immunostaining for phospho-τ (AT8) showed prominent τ-astrogliopathy affecting the cornu ammonis subregions and the dentate gyrus (Figure 1F, G); in sum reminiscent of that described in a subset of elderly individuals [4, 5]. CA was present also related to τ-positive astrocytic processes (Figure 1G, right upper inset). CA was prominent in the cerebellum also where it associated with τ-pathology (Figure 1H). 

The conditions that favor the development of CA, include aging, neurodegeneration, chronic hypoxia, and diabetes mellitus [6]. CA is thought to be mostly of astrocytic origin and their development is considered to be triggered by cellular injury [1, 7]. Although in our case we cannot trace the etiology of the abundant CA or their relation to clinical symptoms, it is of note that the CA associated with intensive τ-astrogliopathy and with TDP-43 proteinopathy. Our observation further emphasizes the need of ongoing neuropathological studies to understand the spectrum of pathologies in the human brain. 

## Conflict of interest 

The authors report no conflict of interest.

**Figure 1:**
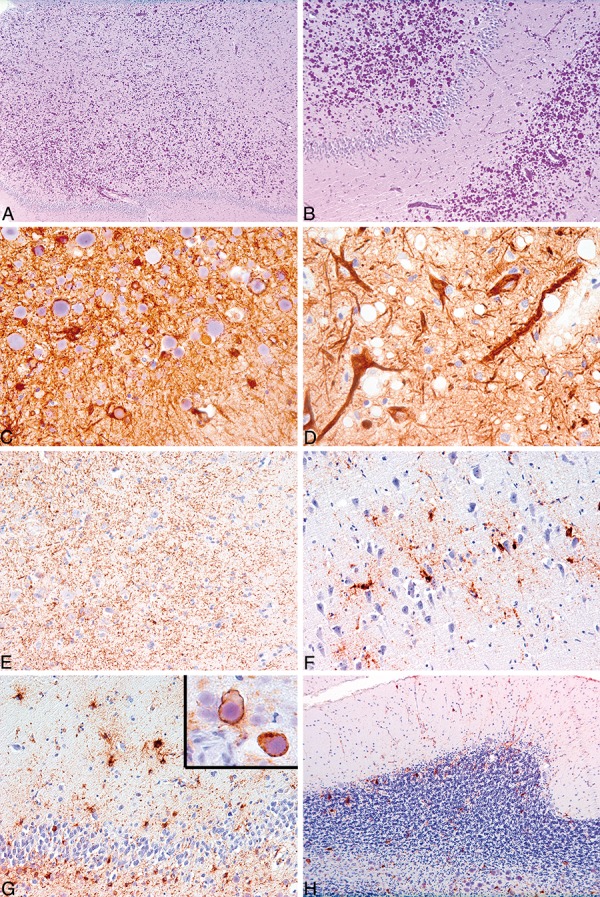
Clinical Neuropathology image 6-2014: Corpora amylacea replacing cornu ammonis (CACA).
